# Spatio-temporal dynamics of human-induced carbon emissions in Southeast Asia (1992–2022) based on nighttime light

**DOI:** 10.1016/j.eehl.2025.100150

**Published:** 2025-04-26

**Authors:** Chaoqing Huang, Qian Wu, Yujie Chen, MinhThu Nguyen, Bin Chen, Song Hong, Chao He

**Affiliations:** a School of Resource and Environmental Sciences, Wuhan University, Wuhan 430079, China; b Key Laboratory of Geographic Information System, Ministry of Education, Wuhan University, Wuhan 430079, China; c School of Electronic Information and Communications, Huazhong University of Science and Technology, Wuhan 430074, China; d Vietnam Institute of Meteorology Hydrology and Climate Change, Ministry of Natural Resources and Environment, Hanoi 100803, Viet Nam; e Future Urbanity & Sustainable Environment (FUSE) Lab, Division of Landscape Architecture, Department of Architecture, Faculty of Architecture, The University of Hong Kong, Hong Kong 999077, China; f National Science Library (Wuhan), Chinese Academy of Sciences, Wuhan 430071, China; g Collaborative Innovation Center for Emissions Trading System Co-constructed by the Province and Ministry, Wuhan 430205, China

**Keywords:** Carbon emissions, Human induced, Nighttime light, Southeast Asia, Spatio-temporal dynamics

## Abstract

Understanding regional carbon emissions from human activities, particularly their spatio-temporal patterns, is essential for implementing decarbonization strategies and cultivating a low-carbon economy. This study develops a spatial visualization model to estimate carbon emissions in Southeast Asia using calibrated nighttime light data, with DMSP-OLS (Defense Meteorological Satellite Program Operational Linescan System) and NPP-VIIRS (National Polar-orbiting Partnership Visible Infrared Imaging Radiometer Suite) standardized through polynomial regression and machine learning to ensure consistency. Emissions in Southeast Asia increased by 2.51 times from 1992 to 2022, shifting from gradual to rapid growth. Validation against Open-source Data Inventory for Anthropogenic CO_2_ (ODIAC) and Emissions Database for Global Atmospheric Research (EDGAR) shows strong agreement in high-emission urban areas but discrepancies in low-emission rural regions due to data sparsity and satellite sensor limits. Spatial analysis reveals that major Southeast Asian cities and their peripheries exhibit robust, sustained growth, while rural, less-developed areas show slower trends, highlighting persistent urban-rural disparities. These urban regions demonstrate a “circular economy advantage”, where per-unit-area carbon emissions steadily rise in economically advantageous zones. Despite high model accuracy, uncertainties persist due to variations in regional economic activities and the limitations of satellite-based emission proxies. Forecasts suggest elevated emission levels in major cities will continue, while changes in other areas remain relatively minimal. Consequently, achieving a low-carbon economy in Southeast Asia requires a top-down approach, emphasizing infrastructure enhancement, resource and energy optimization, and fostering a sustainable, circular socio-economic system.

## Highlights

• Calibrated DMSP and VIIRS nighttime light data to a common scale using a nonlinear model.
• Developed a spatial visualization model for carbon emissions integrating nighttime light, economic, land cover, and annual emission data.
• Found a 2.51-fold increase in carbon emissions in Southeast Asia from 1992 to 2022, with major cities showing high and sustained growth.

## Graphical abstract

**Figure:**
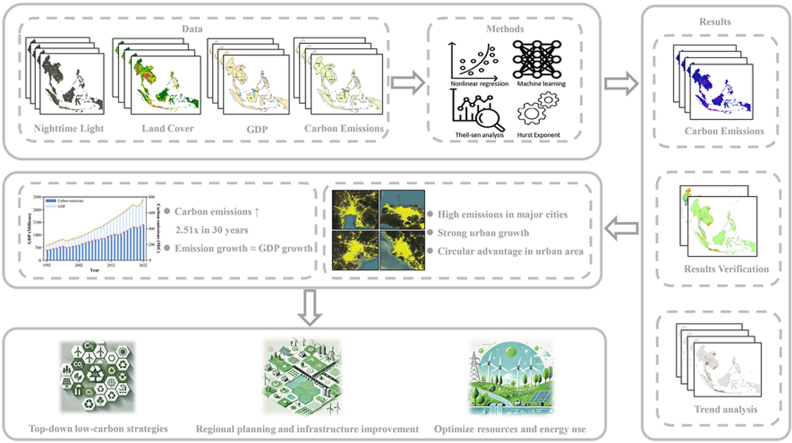

## 1 Introduction

Over the past century, continuous advancements in societal and technological activities have significantly increased carbon dioxide emissions. The rise in greenhouse gas concentrations has triggered widespread climate change [[1], [2], [3]]. The consequences of global climate change, including rising sea levels, increased frequency of extreme weather events, and ecosystem collapse, pose significant threats to human society [4,5]. As a result, various entities, such as the United Nations, member states, and international organizations, are actively advocating for strategies to mitigate carbon emissions and address the challenges of global climate change. As a central institution for global climate action, the United Nations promotes international cooperation mechanisms like the United Nations Framework Convention on Climate Change (UNFCCC) and the Paris Agreement. The IPCC periodically publishes global climate assessment reports, providing the latest scientific knowledge and data to support policymakers. The International Energy Agency (IEA) proposes diverse energy transition and decarbonization pathways to achieve sustainable energy development and carbon neutrality goals. Furthermore, governments and regional organizations have developed specific emission reduction plans. For instance, the European Union has introduced the “Green Deal” to position Europe as a global leader in the low-carbon economy [6]. China has set ambitious targets to peak carbon dioxide emissions by 2030 and achieve carbon neutrality by 2060, focusing on renewable energy, clean transportation, and low-carbon cities [7]. In 2015, Southeast Asian member states adopted the “ASEAN Low Carbon Development Blueprint 2025” to stimulate low-carbon economic development in the region [8].

Understanding the spatio-temporal patterns of regional carbon emissions is crucial for the effective implementation and long-term operation of carbon reduction plans. However, examining these patterns on a regional scale has traditionally required significant resource coordination due to limitations in accounting methods and monitoring techniques. Traditional bottom-up inventory-based approaches accurately depict carbon emissions from human activities at various scales. However, they are typically labour-intensive, time-consuming, and inefficient, posing challenges in spatially representing the statistical outcomes [[9], [10], [11]]. The successful use of carbon satellite data in Japan, the United States, and China has partially resolved the delay in carbon emission monitoring and enhanced monitoring efficiency. However, carbon satellites are constrained by natural conditions such as atmospheric circulation and soil carbon emissions, which can lead to less reliable monitoring outcomes. Additionally, carbon satellite retrievals provide atmospheric column concentrations of carbon dioxide, necessitating further exploration of how to use this data to estimate regional human activity carbon emissions [[12], [13], [14], [15]]. Alternatively, station observations or sensor monitoring of carbon concentrations has progressively improved. However, these methods are costly, have limited coverage, and are more suitable for verification when monitoring carbon emissions on more minor scales [16,17].

With the advancement of remote sensing and computer technology, numerous researchers have utilized remote sensing and statistical data to analyze and model carbon emissions across various scales and spatio-temporal patterns. Su et al. evaluated carbon dioxide emissions from energy consumption in Chinese cities using Defense Meteorological Satellite Program Operational Linescan System (DMSP-OLS) nighttime light (NTL) images. This study scrutinized the spatio-temporal dynamics of carbon emissions in China over 19 years, identified the main driving factors of carbon emissions in different regions, and proposed corresponding mitigation policies [18]. Zhao et al. integrated two NTL datasets from 1992 to 2016 ​at the pixel level, estimated the spatio-temporal distribution of carbon emissions based on regression models, and analyzed the dynamics and driving forces of carbon emissions from urban residents in China [19]. Lv et al. fitted the relationship between DMSP-OLS data and National Polar-orbiting Partnership Visible Infrared Imaging Radiometer Suite (NPP-VIIRS) composite data to obtain accurate results, constructing a model to estimate carbon emissions using NTL data from 1995 to 2016 alongside carbon emission statistics. This model analyzed the spatio-temporal trends of carbon emissions in China at four scales: pixel, province, region, and county [20]. Chen et al. used modified NPP-VIIRS NTL data to reexamine and verify the environmental Kuznets curve for carbon emissions in Chinese cities, aiming to more accurately measure carbon emissions at the urban scale and identify patterns of urban-scale carbon emissions, thus providing references for decision-making [21]. Yang et al. estimated urban-scale carbon emissions using the relationship between DMSP-OLS NTL data and carbon emissions, analyzing the spatio-temporal patterns of carbon emissions in the three northeastern provinces of China from 1998 to 2013 [22]. Building on this approach, Wang et al. [23] integrated multisource remote sensing data, including NTL and column-averaged CO_2_ dry-air mole fraction (XCO_2_) observations, to improve carbon emission estimation accuracy, while Wang et al. [24] developed an XCO_2_ anomaly-based method to enhance the detection of anthropogenic carbon emissions using Open-source Data Inventory for Anthropogenic CO_2_ (ODIAC) and temperature data, providing new insights into satellite-based emission monitoring. This study demonstrated that NTL imagery is a novel and effective method for monitoring urban-scale carbon emissions. Compared to traditional IPCC carbon accounting methods, this approach offers advantages in low manual cost, speed, and efficiency [22].

From 1990 to 2020, Southeast Asia underwent rapid industrialization, with GDP growth averaging 4.5% annually over the past three decades [25]. The share of fossil fuels in Southeast Asia's energy consumption increased from 53% to 84.86%, indicating a rapid and sustained growth trend (https://www.iea.org). This trend demonstrates the dominant position of fossil fuels in the region's energy consumption, which is becoming increasingly pronounced. The ASEAN Low Carbon Development Blueprint aims to reduce carbon intensity by 30% by 2030, necessitating improved emission tracking and targeted policy interventions [26]. Using NTL to infer the spatio-temporal patterns of carbon emissions from fossil fuel consumption in Southeast Asia is fundamental for advancing regional carbon reduction efforts. Gaughan et al. assessed NTL and population distribution to map human-induced carbon emissions in Vietnam, Cambodia, and Laos. The study found that combining NTL with population distribution provided more accurate carbon emission estimates than using each indicator alone. This accuracy improved over time due to the expansion of nighttime lighting areas associated with the development. Furthermore, using multiple data sources offers a more comprehensive understanding of regional emission patterns than relying on a single data source [27]. Oda et al. produced a global high-resolution gridded emission dataset known as ODIAC 2016. This dataset was developed by compiling profiles of power plants, including emission intensities and geographic location information. This information was then combined with satellite-observed NTL to estimate carbon emissions on land at a spatial resolution of 1 ​km ​× ​1 ​km. The results were validated using other datasets, demonstrating that the ODIAC 2016 dataset provides more accurate and detailed information on carbon emissions than previous methods [28,29]. The Joint Research Centre (JRC) of the European Commission has compiled a comprehensive global database of greenhouse gas and air pollutant emissions [30,31]. This database includes carbon emissions data from various sources, such as national inventories submitted to the UNFCCC, energy statistics, and other relevant data sources. We process the data using a combination of bottom-up and top-down approaches and provide it as gridded data with a resolution of 0.1° ​× ​0.1°, covering annual carbon emissions from 1970 to the present.

Our literature comparison analysis revealed a lack of research on the inversion of carbon emissions and related indicators at different spatio-temporal scales using NTL and other auxiliary data. Moreover, most existing studies have focused primarily on China. Extensive research by numerous Chinese scholars has confirmed the close relationship between NTL and carbon emissions at various scales in China, revealing distinct regional characteristics and patterns in their spatio-temporal distribution. These findings have laid a theoretical foundation for reducing carbon emissions and promoting clean energy use. Studies have also been on global-scale human-induced carbon emissions, combining NTL with other data sources such as ODIAC and Emissions Database for Global Atmospheric Research (EDGAR). However, research on regions outside China at a regional scale remains relatively scarce. Southeast Asia, a developing area, has made significant socio-economic progress over the past few decades. However, this progress has been accompanied by extensive fossil energy consumption, making Southeast Asia an increasingly important source of international carbon emissions. Southeast Asian countries are gradually promoting carbon emission reduction to achieve sustainable development in ecology, environment, and socio-economy. Thus, comprehensively understanding the spatio-temporal patterns and variations of human-induced carbon emissions in Southeast Asia is crucial for implementing effective carbon emission reduction strategies. In this study, we generated calibrated NTL data from 1992 to 2022 by fitting DMSP-OLS and NPP-VIIRS products. We then constructed a regional estimation model for human-induced carbon emissions by analyzing NTL, economic, and carbon emission data. Using this model, we estimated human-induced carbon emissions in Southeast Asia over 31 years, exploring and summarizing the spatio-temporal patterns and trend variations of these emissions. This study fills the gap in understanding the spatio-temporal patterns and evolution of human-induced carbon emissions in Southeast Asia, providing data and theoretical support for carbon emission reduction policy.

## 2 Data and methods

### 2.1 Study area

**Fig. 1:**
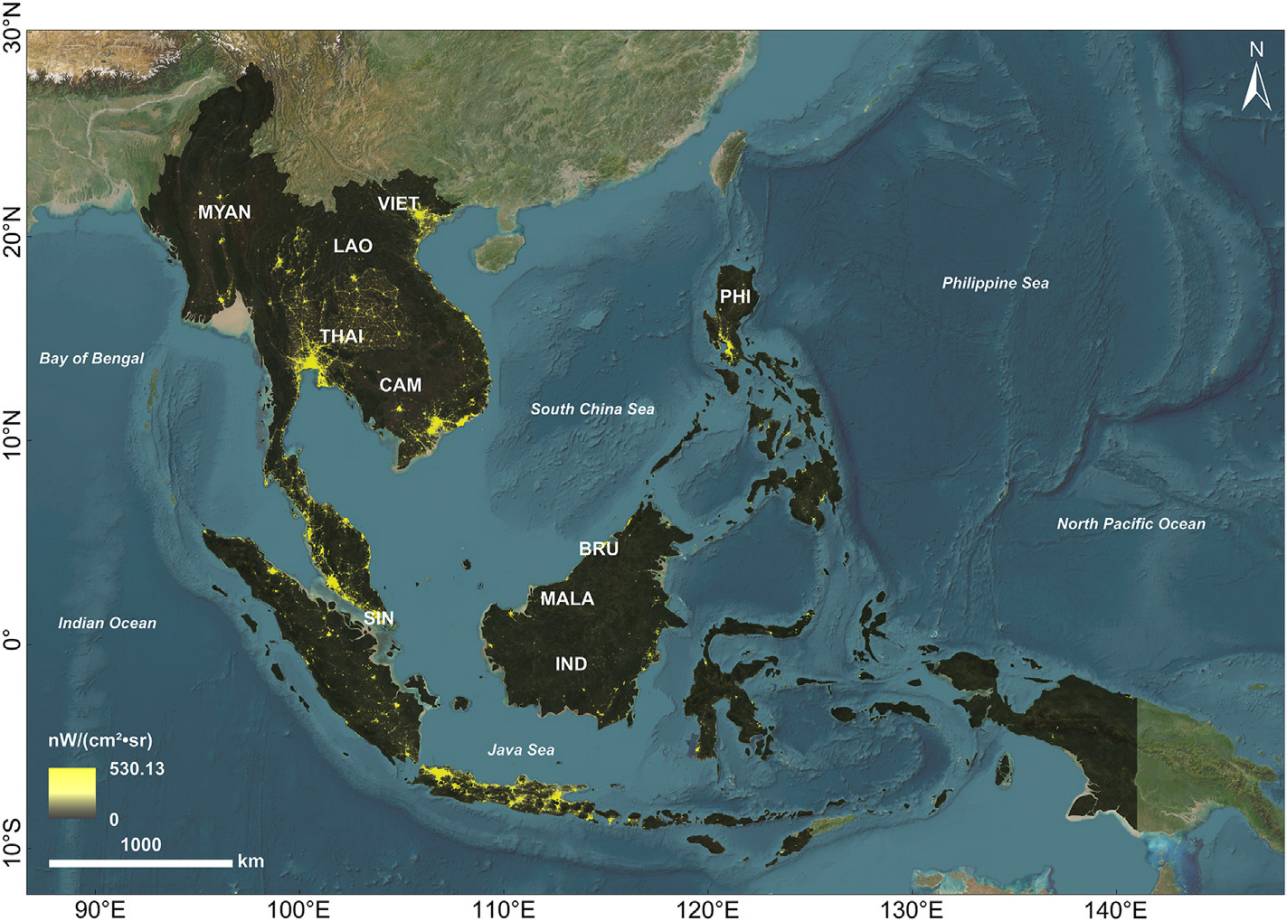
Study area map of Southeast. BRU, Brunei; CAM, Cambodia; INDO, Indonesia; LAO, Laos; MALA, Malaysia; MYAN, Myanmar; PHI, The Philippines; SIN, Singapore; THAI, Thailand; VIET, Vietnam.

Southeast Asia lies between 92°E and 140°E longitude and between 10°S and 28°26′N latitude. China borders the region to the north, the Pacific Ocean to the east, the Indian Ocean to the west, and the sea separates it from Australia to the south. Southeast Asia comprises the countries of Brunei (BRU), Cambodia (CAM), Indonesia (INDO), Laos (LAO), Malaysia (MALA), Myanmar (MYAN), The Philippines (PHI), Singapore (SIN), Thailand (THAI), and Vietnam (VIET). Encompassing an area of approximately 4.49 million square kilometres (Fig. 1), Southeast Asia had a population of around 673.66 million people in 2022. Between 1992 and 2022, the region's GDP increased 3.03 times, while human-induced carbon emissions rose significantly, indicating substantial developmental trends.

### 2.2 Data and preprocessing

The data used in this study include NTL data, human-induced carbon emission data, economic data, land cover data, and high-resolution remote sensing images. The NTL data were obtained from two distinct satellite remote sensing data series: DMSP-OLS sensor data, which covers 1992 to 2013, and NPP-VIIRS sensor data, which spans from 2013 to 2022. Data from these five sensors were processed for smoothing and resolution to create cloud-free composites [32,33]. The NPP-VIIRS sensor provides atmospherically calibrated NTL products based on the bidirectional reflectance distribution function (BRDF) [34]. Human-induced carbon dioxide emission data were sourced from various organizations, including the IEA, the ODIAC, the EDGAR, and the World Bank. Economic data were obtained from the World Bank and the International Monetary Fund (IMF), while land cover data were extracted from the Moderate Resolution Imaging Spectroradiometer (MODIS) annual land cover products. High-resolution images were acquired from Google Earth. Additional details about the datasets can be found in Table S1.

The Google Earth Engine (GEE) platform was used to acquire NTL and land cover data (Fig. S1), which were then resampled to a grid resolution of 1 ​km using the WGS1984 spatial reference system. Geographic Information System (GIS) tools were utilized to process the ODIAC and EDGAR data products, ensuring consistent spatial resolution and reference.

### 2.3 Methods

#### 2.3.1 Calibration of NTL data

This research calibrated NPP-VIIRS NTL data using DMSP-OLS data to establish a consistent NTL dataset for Southeast Asia spanning 1992 to 2022. The DMSP-OLS data covers 1992 to 2013, while the NPP-VIIRS dataset extends from late January 2012 onwards. The DMSP-OLS data provides dimensionless relative brightness values ranging from 0 to 63. In contrast, the NPP-VIIRS data delivers dimensional values ranging from 0 to several hundred or thousand nanowatts per square centimetre per steradian [nW/(cm^2^·sr)]. We assessed the correlation between these two NTL datasets during their overlapping years. The greater sensitivity of NPP-VIIRS data allows for more accurate retrieval of high-intensity light sources. In contrast, the narrower intensity range of DMSP-OLS data may lead to saturation issues. Previous studies have employed polynomial regression [35] and quantile-based normalization [36] to reconcile these differences; however, these methods may not fully account for regional variations in illumination patterns. To improve calibration accuracy, we adopted multiple regression models, including Linear, Polynomial, Exponential, Random Forest, Support Vector, and K-Nearest Neighbors Regression, to adapt NPP-VIIRS data to a range comparable with the DMSP-OLS threshold (Table S2). Compared to previous methods, our approach integrates machine learning techniques (Random Forest, Support Vector Regression), which enhance adaptability to spatial heterogeneity and reduce saturation bias in high-illumination zones. We also compared the unified NTL dataset with the methods and accuracy metrics reported by Yu et al. [37], thereby validating the reliability of our calibration results. Additionally, we used the MCD12Q1 land cover dataset to standardize the range of these two NTL datasets. We extracted built-up areas for 2013 and used these areas to truncate the DMSP-OLS and NPP-VIIRS data for the same year. This step minimizes the influence of background noise from non-urban areas, thereby refining the calibration accuracy.

#### 2.3.2 Construction of carbon emission estimation model using NTL and economic data

In examining the correlation between Southeast Asia's NTL and total human activity-related carbon emissions for corresponding years, we found a strong relationship between the average and total values of NTL's digital number (DN) and human-induced carbon emissions. This relationship was expressed as a linear equation, with an R^2^ of 0.770 (*p* ​< ​0.05) for the mean DN of NTL and carbon emissions and an R^2^ of 0.765 (*p* ​< ​0.05) for the total DN of NTL and carbon emissions. Given the close association between human activity-related carbon emissions and socio-economic development, we integrated economic data into our analysis. Consequently, we conducted an exploratory study using the average DN and total DN of Southeast Asia's NTL from 1992 to 2022, GDP data, and total carbon emissions data. This study aimed to establish a model for estimating human activity-related carbon emissions based on NTL data and economic indicators.

#### 2.3.3 Grid-based calculation of human activity carbon emissions

Carbon emissions corresponding to each DN value in the annual NTL data of Southeast Asia were calculated using Eq. 1.

(1) $${{DN}_{\_unit}=HCE}_{\_total}/{NTL}_{\_total}$$

where ${DN}_{\_unit}$ denotes the carbon emissions corresponding to each DN value in the carbon emissions grid data for a given year, ${HCE}_{\_total}$ signifies the total human carbon emissions in the region for that year, and ${NTL}_{\_total}$ represents the total DN value in the NTL data for that year.

The grid-based scenario of human activity carbon emissions in Southeast Asia was determined using Eq. 2.

(2) $${{HCE}_{\_ras}=DN}_{\_value}\times {DN}_{\_unit}$$

where ${HCE}_{\_ras}$ signifies the spatial grid data of human activity carbon emissions in Southeast Asia for a specific year after grid-based processing. ${DN}_{\_value}$ stands for the DN value of each grid in the NTL for that year, and ${DN}_{\_unit}$ corresponds to the carbon emissions per DN value in the carbon emissions grid for that year.

#### 2.3.4 Machine learning-based regression for NTL calibration

This study employs Random Forest Regression (RFR) and Support Vector Regression (SVR) to model the nonlinear relationship between NPP-VIIRS and DMSP-OLS NTL data using the overlapping 2013 dataset, aiming to establish a reliable calibration framework. RFR, an ensemble learning method, enhances prediction stability by aggregating multiple decision trees, making it well-suited for high-dimensional remote sensing data [38]. SVR constructs a hyperplane in high-dimensional space to minimize prediction errors while maintaining model generalizability, which is particularly effective for heterogeneous data distributions [39]. These models are trained to predict DMSP-OLS-equivalent values from NPP-VIIRS data, ensuring temporal consistency in long-term NTL datasets. Model performance is evaluated using R^2^ and RMSE, confirming that machine learning-based calibration improves dataset continuity and enhances the accuracy of NTL-based carbon emission estimations [40].

#### 2.3.5 Temporal and spatial analysis of human activity carbon emissions

We used the Theil-Sen Median estimator and the Mann–Kendall (M−K) test to calculate the trend of changes in the DN values of human activity-related carbon emissions data from 1992 to 2022 in Southeast Asia. The Theil-Sen estimator and M−K test are non-parametric methods suitable for analyzing non-normally distributed datasets. Unlike parametric regression models, these methods do not assume a normal distribution and are robust to outliers. Compared with trend analysis based on the least squares method, the Theil-Sen estimator avoids issues related to the lack of time series data and the influence of data distribution on analysis results, effectively eliminating the interference of outliers in the time series [41].

(3) $$S_{HCE}=median\left[\left({HCE}_{j}-{HCE}_{i}\right)/\left(j-i\right)\right]\;\;\;\;\;\;1<i<j<n$$

where *i* and *j* are the time series numbers, ${HCE}_{i}$ and ${HCE}_{j}$ are human activity carbon emissions data's DN values of the time series in the *i*th year and the *j*th year, respectively. The slope $S_{HCE}$ is greater than 0, indicating an upward trend; when $S_{HCE}$ is less than 0, it means a downtrend. The M−K test does not require samples to follow a particular distribution, which can eliminate a few outliers and is suitable for non-normally distributed data. The inspection process is as Eq. 5.

(4) $$\tau =\sum_{i=1}^{n-1}\sum_{j=i+1}^{n}sign\left({HCE}_{j}{-HCE}_{i}\right)$$

(5) $$sign\left({HCE}_{j}{-HCE}_{i}\right)=\left\{\begin{array}{c} 1\;{\;\;\;\;\;\;\;\;\;\;\;\;\;\;\;HCE}_{j}-{HCE}_{i}>0\\ 0\;{\;\;\;\;\;\;\;\;\;\;\;\;\;HCE}_{j}-{HCE}_{i}=0\\ -1\;{\;\;\;\;\;\;\;\;\;\;\;HCE}_{j}-{HCE}_{i}<0 \end{array}\right\}$$

Statistics for constructing trend analysis tests use Eqs. (6), (7).

(6) $$U_{MK}=\left\{\begin{array}{c} \left(\tau -1\right)/{\left[Var\left(\tau \right)\right]}^{1/2}\;\;\;\;\;\;\;\;\;\;\;\;\;\;\;\;\;\;\;\;\;\;\;\;\;\;\tau >0\\ 0\;\;\;\;\;\;\;\;\;\;\;\;\;\;\;\;\;\;\;\;\;\;\;\;\;\;\;\;\;\;\;\;\;\;\;\;\;\;\;\;\;\;\;\;\;\;\;\;\;\tau =0\\ \left(\tau +1\right)/{\left[Var\left(\tau \right)\right]}^{1/2}\;\;\;\;\;\;\;\;\;\;\;\;\;\;\;\;\;\;\;\;\;\;\;\;\;\;\tau <0 \end{array}\right\}$$

(7) Var(τ)=n(n−1)(2n+5)/18

where τ is the M−K rank correlation coefficient, measuring the direction and strength of the trend; $U_{MK}$ is the standardized M−K statistic used to test the significance of the trend; *n* represents the time series length; the *sign* is a symbolic function. At a given significance level α, $\left|U_{MK}\right|>u_{1-\alpha /2}$ indicates a statistically significant trend, where $u_{{}_{1-\alpha /2}}$ is the critical value from the standard normal distribution used to evaluate the significance of $U_{MK}$.

The Hurst exponent is one of the effective methods to quantitatively describe long-term dependencies within time series [42,43]. This paper describes the future trend of the total DN value of carbon emissions data. The basic principle is:

For the time series $\left\{{HCE}_{\left(t\right)}\right\},t=1,2,\ldots ,n$, define the mean series as:

(8) $$\bar{{HCE}_{\left(T\right)}}=\left(1/T\right)\sum_{t=1}^{T}{HCE}_{\left(t\right)},T=1,2,\ldots ,n$$

(9) $$\text{Accumulated}\;\text{deviation}:{\;X}_{\left(t,T\right)}=\sum_{t=1}^{t}\left({HCE}_{\left(t\right)}-\bar{{HCE}_{\left(T\right)}}\right)$$

(10) $$\text{Range}:R_{\left(t\right)}=\max_{1\leq t\leq T}X_{\left(t,T\right)}-\min_{1\leq t\leq T}X_{\left(t,T\right)}$$

(11) $$\text{Standard}\;\text{deviation}:S_{\left(T\right)}={\left[\left(1/T\right)\sum_{t}^{T}{\left({HCE}_{\left(t\right)}-{HCE}_{\left(T\right)}\right)}^{2}\right]}^{1/2}$$

In the formula: 1≤t≤T,T=1,2,……,n; calculate the ratio according to the range and standard deviation $R_{\left(t\right)}/S_{\left(T\right)}≌R/S$, if $R/S\propto T^{H}$, indicating a Hurst phenomenon in the analysis of the total DN value of carbon emissions within a time series, and *H* is the Hurst exponent. When *H* ​= ​1/2, it indicates that the future of the sequence has nothing to do with the past; when *H* ​> ​1/2, it suggests that the future trend is consistent with the past, that is, the process is persistent, and the closer *H* is to 1, the stronger the continuity; When *H* ​< ​1/2, it indicates that the future trend is opposite to that of the past, that is, the process is anti-persistent, and the closer H is to 0, the stronger the anti-persistence is.

We conducted pixel-level spatial correlation analysis [44] to examine the spatial relationship between our research findings and the ODIAC and EDGAR data products. The formula for calculating the spatial correlation coefficient is as follows:

(12) $$R_{xy}=\frac{n\sum_{i=1}^{n}\left(X_{i}-\bar{X}\right)\left(Y_{i}-\bar{Y}\right)}{\sqrt{\sum_{i=1}^{n}{\left(X_{i}-\bar{X}\right)}^{2}}\sqrt{\sum_{i=1}^{n}{\left(Y_{i}-\bar{Y}\right)}^{2}}}$$

where $R_{xy}$ represents the correlation coefficient between our research findings and the ODIAC or EDGAR data products. $X_{i}$ and $Y_{i}$ represent the carbon emissions of our research findings and the ODIAC (or EDGAR) data products for the *i*th year, respectively. $\bar{X}$ and $\bar{Y}$ denote the multi-year averages of the two variables, and *n* represents the number of years.

### 2.4 Study flow chart

Our study involved calibrating Southeast Asia's 2013 DMSP-OLS data to align with the NPP-VIIRS data, using 2013 land cover data for verification to ensure consistency in built-up area representation, resulting in a calibration model for NTL data. Using this model, we calibrated the NPP-VIIRS NTL data from 2014 to 2022, creating a harmonized NTL data from 1992 to 2022 based on the DMSP-OLS standard. The calibrated NTL data was combined with economic data and human-induced carbon dioxide emissions from Southeast Asia for data analysis and fitting, enabling us to derive a model for estimating carbon dioxide emissions in the area. We computed the anthropogenic carbon emissions in Southeast Asia from 1992 to 2022 using the mean and total DN values of the calibrated NTL data and the annual GDP data of the region. We verified the reliability of our model by comparing it with statistical data from institutions such as the IEA, the World Bank, ODIAC, and EDGAR. Additionally, we conducted a spatio-temporal pattern analysis to examine carbon emissions in Southeast Asia from 1992 to 2022. The study flow chart is presented in Fig. S2.

## 3 Results

### 3.1 Calibration of Southeast Asia nighttime light data

In this study, we compared linear, polynomial, exponential, logarithmic fitting, and machine learning models (Random Forest, Support Vector, and K-Nearest Neighbors Regression) for the 2013 DMSP-OLS and NPP-VIIRS NTL data. The exponential regression model achieved the highest accuracy among these models, with an R^2^ value of 0.86 and a significance level of *p* ​< ​0.05, making it the most effective calibration method. The logarithmic and machine learning-based models also performed well, with R^2^ values of 0.80 (logarithmic regression), 0.80 (Random Forest), 0.85 (Support Vector Regression), and 0.83 (K-Nearest Neighbors Regression), indicating their suitability as alternative approaches. The polynomial regression model exhibited moderate performance with an R^2^ value of 0.46. In contrast, the linear regression model had the lowest accuracy, achieving an R^2^ value of only 0.33, suggesting that a simple linear relationship does not adequately capture the complexity of the calibration process. In all models, the relationship between the DN values of DMSP-OLS (y) and NPP-VIIRS (x) remained statistically significant at *p* ​< ​0.05.

We used an exponential model to calibrate the NPP-VIIRS NTL data to align with the DMSP-OLS data threshold range. The calibrated images effectively preserved the detail of the NPP-VIIRS light data while expanding the overall light range in high-intensity regions' surrounding areas, such as major cities, due to DN value saturation (Fig. S3). Additionally, some non-light areas in the NPP-VIIRS data, such as the central part of Kalimantan Island, exhibited impurities due to atmospheric scattering and other factors. Although these impurities were enhanced after model calibration, the distinction between light and non-light areas remained clear.

### 3.2 Nighttime light inversion for estimating Southeast Asia human-induced carbon emissions

Table 1 presents the estimation models derived from fitting the GDP, mean DN value of NTL, total DN value, and carbon emissions for each Southeast Asian member country to the respective year's data. The trilinear equation-based carbon emissions estimation models demonstrate excellent performance, with R^2^ values exceeding 0.90 for all Southeast Asian countries, except for Brunei and Singapore, which have R^2^ values around 0.75. Notably, the significance level of the carbon emissions estimation models for Southeast Asia and its countries is below 0.01. To further validate the robustness of our emission estimation model, we implemented machine learning techniques, including Random Forest and Support Vector Regression. However, as shown in Table S3, the Random Forest and SVR models exhibited comparable accuracy but did not surpass the performance of the trilinear regression models. While machine learning approaches remain valuable, the trilinear equation provides a more stable and interpretable framework for carbon emission estimation in the study region.

**Table 1:** Estimation models for carbon emissions in Southeast Asia and its member countries.

Countries/Regions	Model	R^2^	*p*-value
Southeast Asia	*y* ​= ​34.99 ​+ ​0.15*x*_1_ – 2200.12*x*_2_ – 0.00048*x*_3_	0.99	<0.01
INDO	*y* ​= ​9.304609 ​+ ​0.171649*x*_1_ – 952.444231*x*_2_ + 0.0005*x*_3_	0.95	<0.01
MALA	*y* ​= ​−2.751297 ​+ ​0.210664*x*_1_ – 2002.929869*x*_2_ + 0.006044*x*_3_	0.96	<0.01
VIET	*y* ​= ​−6.1386 ​+ ​0.28601*x*_1_ + 1254.79101*x*_2_ – 0.00368*x*_3_	0.97	<0.01
THAI	*y* ​= ​6.2524497 ​+ ​0.1624061*x*_1_ – 3702.1207764*x*_2_ + 0.0069248*x*_3_	0.95	<0.01
PHI	*y* ​= ​9.809307 ​+ ​0.063876*x*_1_ + 235.666873*x*_2_ – 0.000785*x*_3_	0.95	<0.01
BRU	*y* ​= ​−1.5422 ​+ ​0.217055*x*_1_ – 3187750502.65*x*_2_ + 548855.114102*x*_3_	0.74	<0.05
LAO	*y* ​= ​−1.23956 ​+ ​0.43669*x*_1_ – 0.21099*x*_2_ + 0.00001*x*_3_	0.86	<0.05
CAM	*y* ​= ​0.0943 ​+ ​0.137797*x*_1_ + 309.010655*x*_2_ – 0.00165*x*_3_	0.95	<0.01
MYAN	*y* ​= ​3.718425 ​+ ​0.068752*x*_1_ + 2542.354813*x*_2_ – 0.003531*x*_3_	0.90	<0.01
SIN	*y* ​= ​1.5121 ​+ ​0.0106139*x*_1_ + 699338879.51*x*_2_ – 1262344.54763*x*_3_	0.75	<0.05

*x*_1_ symbolizes the total annual GDP (in trillion US dollars), *x*_2_ signifies the mean DN value of NTL within the estimation range, *x*_3_ denotes the total DN value of NTL, and *y* represents the carbon emissions from human activities, measured in a million metric tons.

**Fig. 2:**
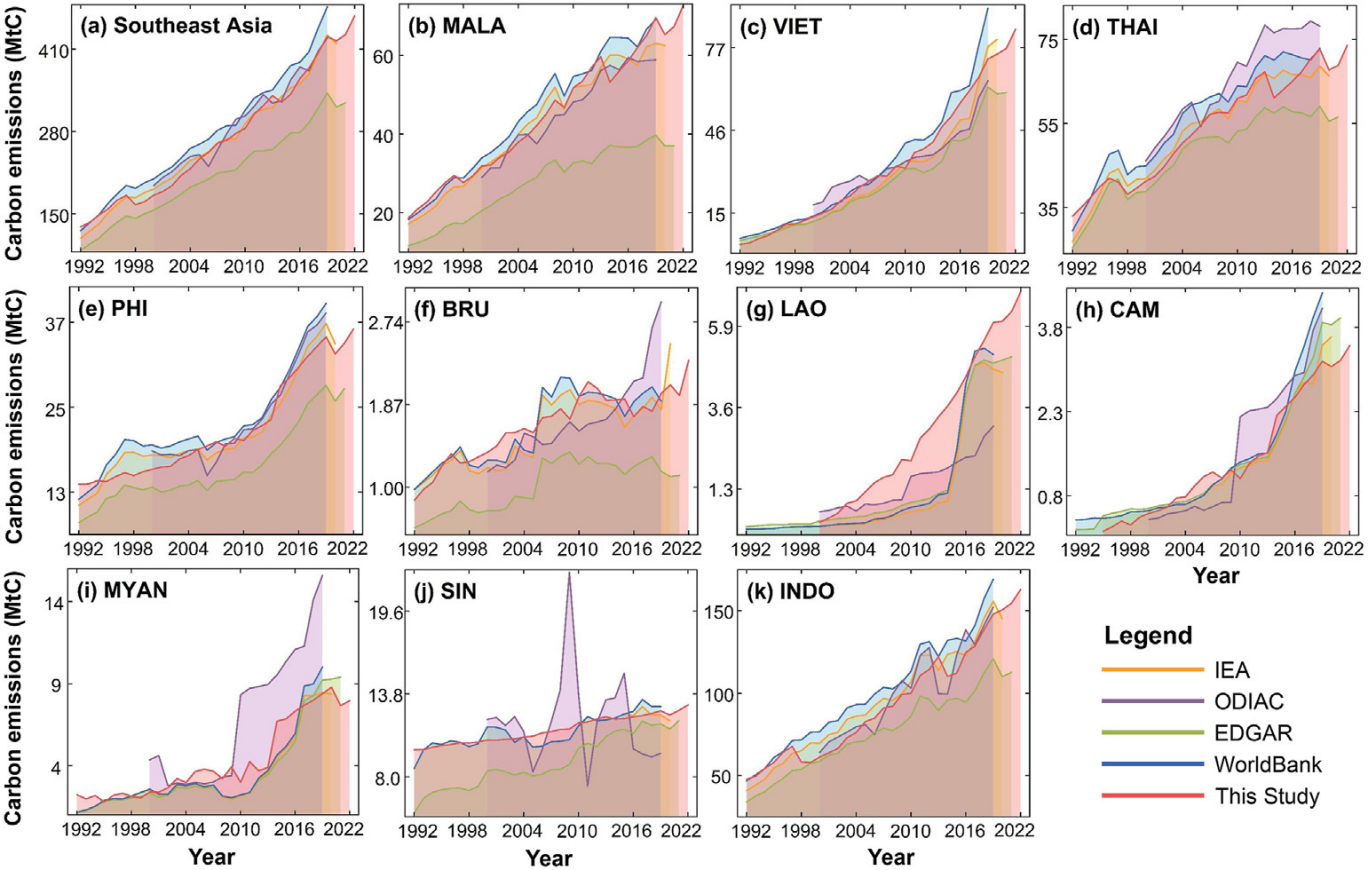
Human-induced carbon emissions of Southeast Asia (a) and its member countries (b–k) from 1992 to 2022.

Our study utilized a carbon emission model integrating NTL and economic data to estimate human-induced carbon emissions across Southeast Asia and its constituent countries from 1992 to 2022. We benchmarked our estimates against statistical data from the IEA, the ODIAC products, the EDGAR, and the World Bank (Fig. 2). The IEA data revealed an increase in Southeast Asia's human-induced carbon emissions from 96.51 MtC in 1990 to 419.03 MtC in 2020, with an average annual emission of 290.62 MtC. ODIAC data from 2000 to 2019 documented an increase from 194.58 MtC to 427.46 MtC, averaging 297.71 MtC annually. EDGAR data from 1992 to 2021 showed growth from 92.16 MtC to 325.36 MtC, with an average annual emission of 233.44 MtC. The World Bank data illustrated an increase from 106.60 MtC in 1990 to 476.75 MtC in 2019, with an average yearly emission of 316.10 MtC. Our study estimated a rise from 128 MtC in 1992 to 449.80 MtC in 2022, with an average annual emission of 282.99 MtC. In terms of average yearly emission growth, the World Bank data recorded the highest increase at 12.34 MtC per year, followed by ODIAC at 11.64 MtC, IEA at 10.40 MtC, our estimates at 10.38 MtC, and EDGAR at 7.77 MtC. Regarding the average annual growth rate, the World Bank data also led at 5.12%, followed by IEA at 4.85%, EDGAR at 4.29%, our findings at 4.14%, and ODIAC at 4.01%. Trends in human-induced carbon emissions in Southeast Asian countries mirrored those of the region, with significant emission increases from 1992 to 2022 in all countries except Singapore and Brunei. The countries with the highest to lowest cumulative emissions were Indonesia (115.25 MtC), Vietnam (80.57 MtC), Malaysia (53.93 MtC), Thailand (40.63 MtC), the Philippines (21.96 MtC), Laos (6.88 MtC), Myanmar (5.75 MtC), Cambodia (3.48 MtC), Singapore (3.16 MtC), and Brunei (1.48 MtC).

**Fig. 3:**
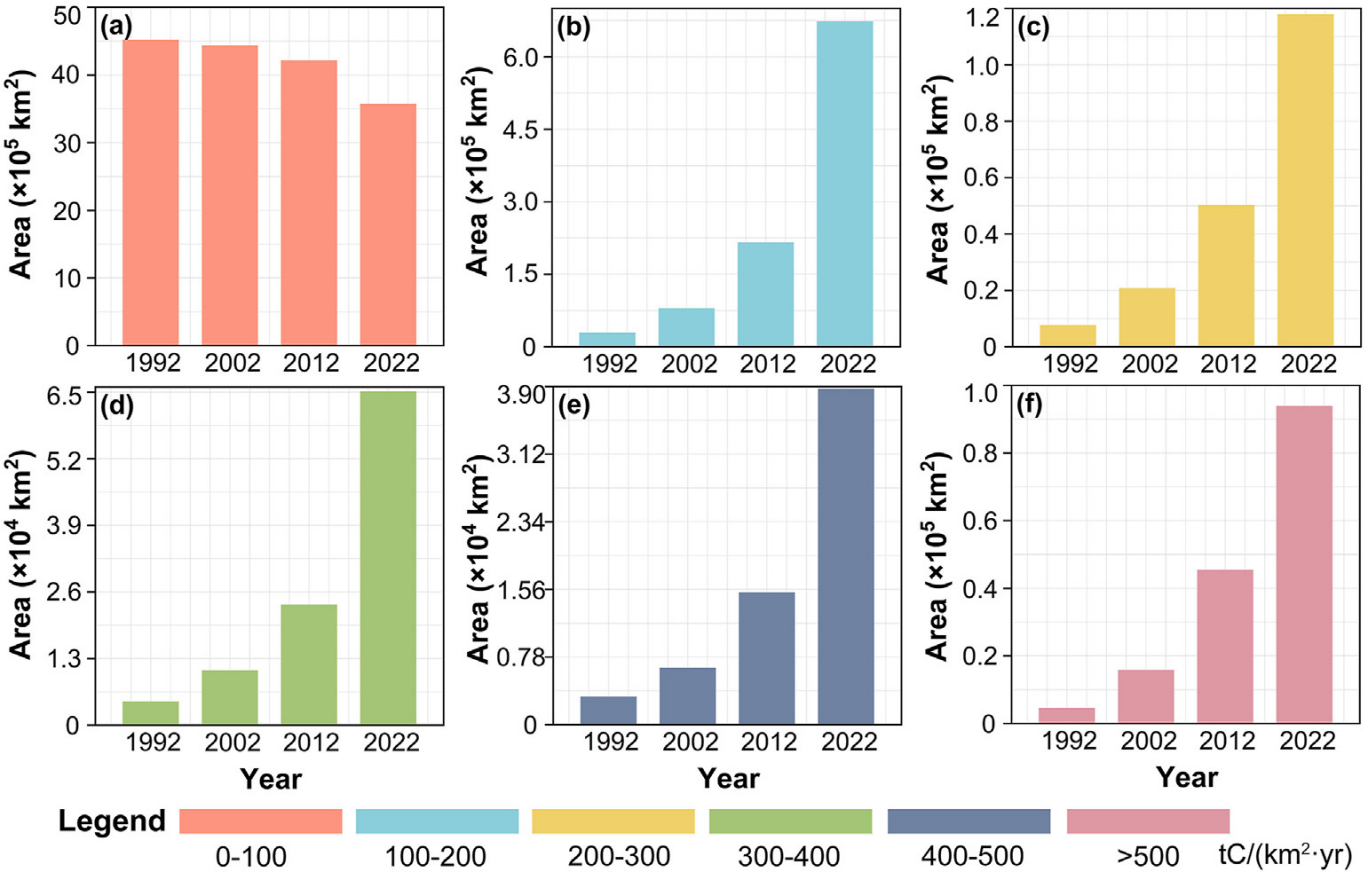
Changes in area with carbon emissions in the ranges of 0–100 (a), 100–200 (b), 200–300 (c), 300–400 (d), 400–500 (e), and >500 tC/(km^2^·yr) (f).

Based on the properties of carbon emissions data, we categorized human-induced carbon emissions per unit area (km^2^) into six levels: 0–100, 100–200, 200–300, 300–400, 400–500, and greater than 500 tC/(km^2^·yr). From 1992 to 2022, we observed significant shifts in the areas covered by each level. The area with carbon emissions in the 0–100 tC/(km^2^·yr) range decreased from 4,514,893 to 3,567,667 ​km^2^ (Fig. 3a), while the area in the 100–200 tC/(km^2^·yr) range expanded from 28,385 to 672,847 ​km^2^ (Fig. 3b). The areas within the 200–300 tC/(km^2^·yr), 300–400 tC/(km^2^·yr), and 400–500 tC/(km^2^·yr) ranges also increased significantly (Fig. 3c–e), as did the area with carbon emissions exceeding 500 tC/(km^2^·yr) (Fig. 3f). Notably, the decade from 2012 to 2022 witnessed a sharp escalation in carbon emissions per unit area, surpassing the emissions observed from 1992 to 2012.

**Fig. 4:**
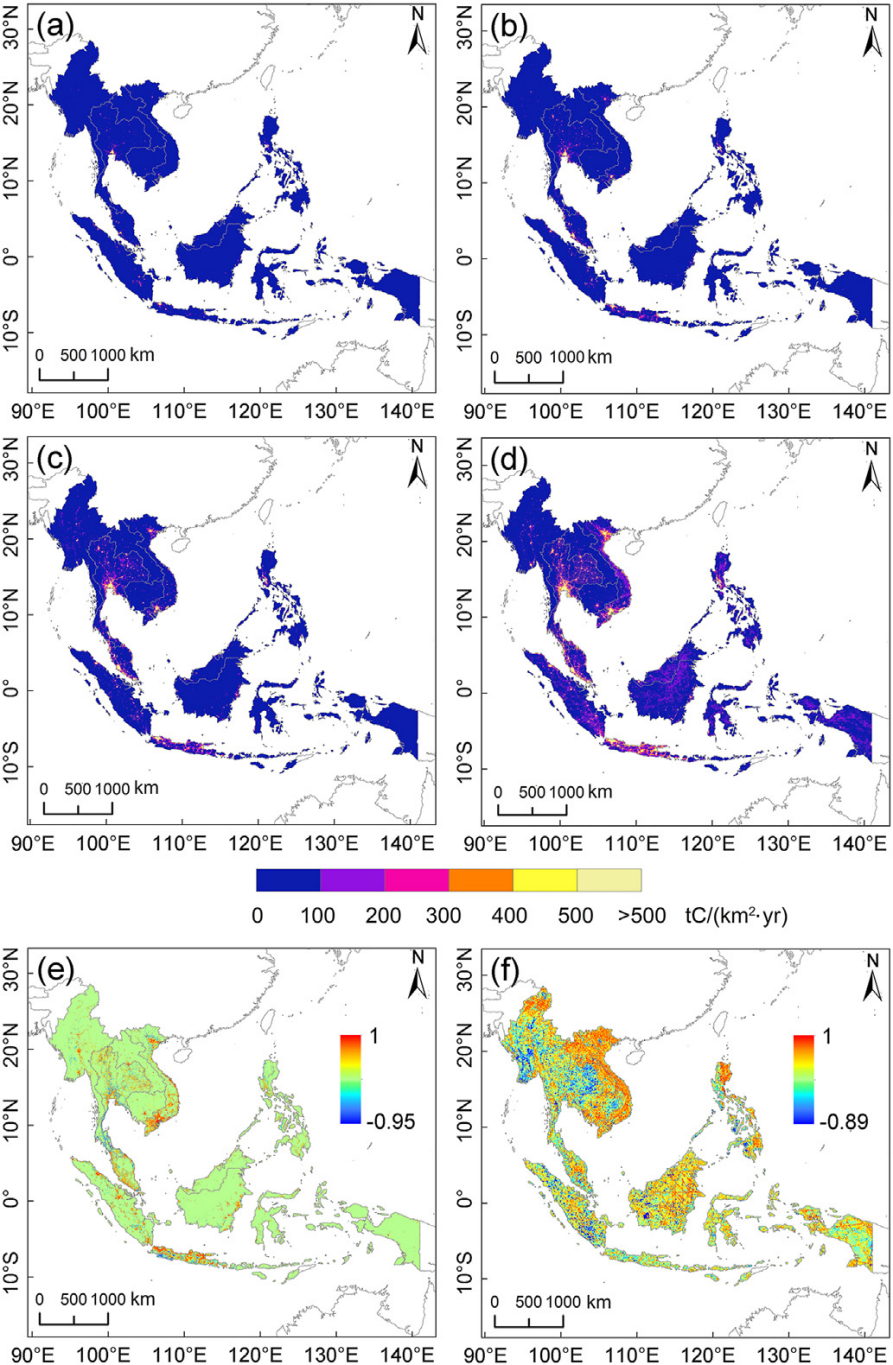
Spatial distribution of carbon emissions in Southeast Asia in 1992 (a), 2002 (b), 2012 (c), and 2022 (d), and validation through correlation with external datasets: ODIAC (e) and EDGAR (f).

Major Southeast Asian cities and their surrounding regions, including Bangkok in Thailand, Ho Chi Minh City and Hanoi in Vietnam, Kuala Lumpur in Malaysia, and Java Island in Indonesia, exhibited significant increases in per unit area carbon emissions from 1992 to 2022. Thailand, Vietnam, Indonesia, and Malaysia also experienced noticeable growth at the national level. Regionally and nationally, the period from 2012 to 2022 witnessed the most significant rise in per unit area carbon emissions, surpassing the changes observed from 1992 to 2002 and 2002 to 2012 (Fig. 4a–d).

We conducted a spatial correlation analysis to examine further the relationship between our research findings and the ODIAC and EDGAR data products. The results showed a high correlation between our findings and the ODIAC data in major Southeast Asian cities and surrounding areas (Fig. 4e). However, the correlation with the EDGAR data was more complex, generally showing a high correlation in the central-southern regions of the Indochinese Peninsula, including Vietnam, Cambodia, northern Myanmar, and the Philippines north, and the vicinity of Kuala Lumpur, Malaysia. In contrast, a negative correlation was observed in southern Myanmar and western Thailand, while other regions demonstrated low or no correlation (Fig. 4f).

Our research findings were compared with those of ODIAC and EDGAR data products. The spatial distribution of human-induced carbon emissions over different years exhibited similar patterns across the three datasets, with pronounced carbon footprints in major Southeast Asian cities and urban areas (Fig. S4). However, in less populated regions such as central Kalimantan in Indonesia, central-southern Laos, and northern Myanmar, our research and the EDGAR data product revealed modest human-induced carbon emissions footprints. At the same time, the ODIAC data suggested near-zero emissions in these areas. From a temporal perspective, all three datasets indicated that the growth rate of human-induced carbon emissions in Southeast Asia from 2010 to 2019 exceeded that from 2001 to 2010.

### 3.3 Spatio-temporal patterns of human-induced carbon emissions in Southeast Asia

**Fig. 5:**
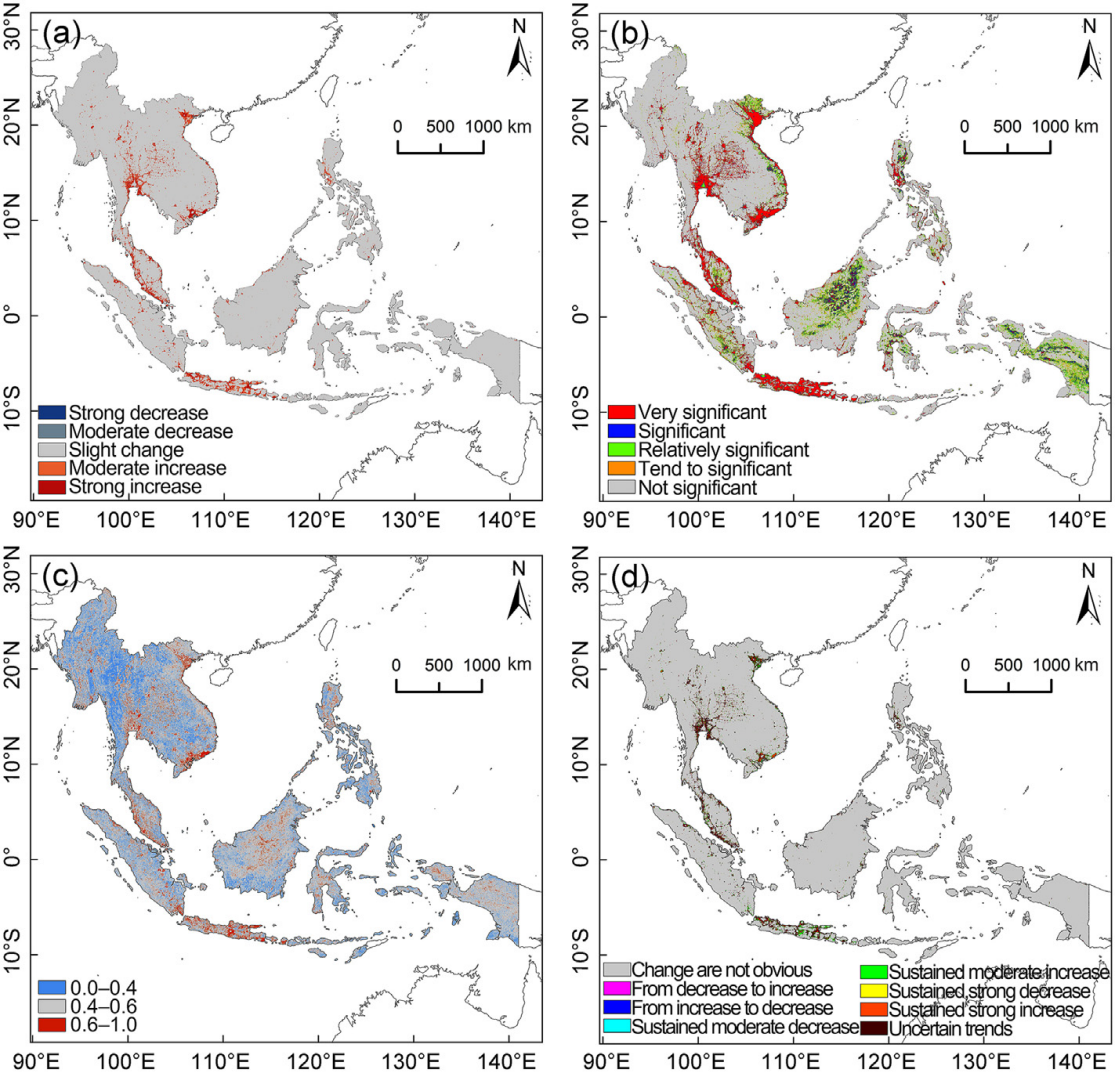
Change trend of human carbon emissions from 1992 to 2022 in Southeast Asia (a), the significance of change trend (b), Hurst exponent (c), and future change trend of human carbon emissions from Southeast Asia (d).

Based on the results of the Theil-Sen estimator, the most dramatic shifts in human-induced carbon emissions in Southeast Asia from 1992 to 2022 were evident in major urban areas and their peripheries, such as Bangkok (Thailand), Hanoi and Ho Chi Minh City (Vietnam), Kuala Lumpur's urban belt (Malaysia), and Java Island's urban clusters (Indonesia). These areas exhibited an escalating trend in carbon emissions, with the strongest surges occurring in city centres and gradually declining to modest increases in the suburbs (Fig. 5a). Excluding sporadic occurrences in the southern region of Sumatra Island, there was scant evidence of a downward trend in human-induced carbon emissions elsewhere. Furthermore, the Mann–Kendall test indicated that areas with the most significant shifts in human-induced carbon emissions from 1992 to 2022 also exhibited the highest levels of statistical significance (Fig. 5b). Only a few areas demonstrated significant or relatively significant trends, while most showed no significance. The Hurst exponent analysis for human-induced carbon emissions from 1992 to 2022 revealed strong persistence in major Southeast Asian urban regions, with future trends likely aligning with past patterns (Fig. 5c). Conversely, regions such as eastern Myanmar, northern Thailand, the southern region of Borneo Island, and the southern Philippines displayed anti-persistence, implying that future carbon emission trends in these areas might diverge from past patterns. In the remaining regions, future carbon emission fluctuations were not correlated with past trends. We investigated the future trends of human-induced carbon emissions in Southeast Asia by integrating the results from the Theil-Sen estimator and Hurst exponent analyses. Significant trend alterations were evident in major urban areas (e.g., Bangkok, Ho Chi Minh City, Hanoi, Kuala Lumpur, Jakarta, etc.) (Fig. 5d). Specifically, future carbon emission trends in the central areas of these cities were less pronounced. In contrast, surrounding areas exhibited a persistent sharp increase and uncertain carbon emission trends. These two regions are spatially intermingled, and the urban fringe areas manifested a consistent moderate increase in carbon emissions. In contrast, future carbon emissions showed unclear trend shifts in the vast areas beyond the cities.

Notably, carbon emissions from human activities in Southeast Asia are predominantly concentrated in major cities and surrounding areas, where industrialization and urban expansion drive significant energy consumption. In contrast, vast rural areas remain underdeveloped, with lower human activity levels and consequently lower carbon emissions, a trend supported by previous studies [45]. Furthermore, as this study estimates emissions using NTL data, the signal intensity in sparsely populated rural areas is minimal, making it challenging to derive meaningful emission estimates. Therefore, this research focuses on urban-centred carbon dynamics, where NTL data provides a reliable indicator of human-induced emissions.

## 4 Discussion

### 4.1 Evaluating the rationality of using multi-year nighttime light data calibration

The DMSP-OLS NTL data, spanning 1992 to 2013, were preprocessed to improve resolution and achieve mutual correction, resulting in consistent, cloud-free files. We then used the DMSP-OLS NTL data threshold to numerically calibrate the NPP-VIIRS (VNP46A4 product) data. Post-calibration, we observed a considerable expansion in the area of high DN values within the NPP-VIIRS NTL data compared to the original data. This expansion is due to the broader range of the NPP-VIIRS NTL threshold, which introduced disparities between low-to-medium DN values and surrounding higher DN value pixels. Calibration amplified the brightness of low-to-medium DN pixels while keeping high DN pixels relatively stable, increasing the illuminated area. The calibrated data closely mirrors the spatial distribution of NPP-VIIRS, particularly in high-emission regions, while extending the low-to-medium DN regions, enhancing spatial coverage.

Despite these improvements, two key uncertainties remain. First, DMSP-OLS data have lower radiometric resolution and are prone to saturation in high-illumination areas, leading to potential underestimation of emissions in dense urban regions. Although calibration reduces discrepancies between DMSP-OLS and NPP-VIIRS, residual saturation effects may still affect emission estimates in megacities. Second, calibration expands low-to-medium DN regions, which improves spatial coverage but may also introduce overestimation in peri-urban and rural areas where weak illumination signals are amplified. Future refinements, such as machine learning-based calibration, could further mitigate these issues and enhance the robustness of emission estimates across different spatial scales.

### 4.2 Examining uncertainty in the spatial distribution of carbon emissions

The spatial consistency between NTL intensity, economic hotspots, and carbon emissions presents an element of uncertainty. While most studies have validated the linear relationship between NTL intensity and local carbon emission density [46,47], the heterogeneity of these two variables suggests that regions with higher carbon emissions do not necessarily exhibit greater NTL intensity [48,49]. Although economic hotspots of human activity are closely associated with the spatial distribution of carbon emissions [50,51], obtaining raster data for these economic hotspots complicates their integration into spatial models. Therefore, regional total carbon emissions are derived by modelling and calculating NTL and financial data, followed by spatial processing based on the linear relationship between NTL and carbon emissions to determine the final spatial distribution of carbon emissions. However, the absence of rasterized economic data may lead to inaccuracies in the spatial representation of carbon emissions. Despite correlation analyses indicating that the simulation results of this study have been well-validated in areas of human activity, we propose that more precise methods are required to accurately depict the spatial carbon emissions of human activities. Such methods include spatializing data from high-emission industrial agglomerations (e.g., power plants, cement plants) over many years and assigning weights for inclusion in carbon emission modelling calculations, yielding more accurate spatial data results for carbon emissions.

We observed a significant correlation between the mean and total DN values of NTL and regional carbon emissions at both regional and national scales, including Southeast Asia. However, due to the limited explanatory power of NTL attributes in capturing spatial variations in human-induced carbon emissions, we have incorporated economic activity data (e.g., GDP) into our modelling analysis. We successfully integrated NTL data with financial data to estimate human-induced carbon emissions by formulating a trivariate linear equation. These results confirm that models based on NTL and economic data can accurately infer the spatial distribution of carbon emissions from human activities in Southeast Asia at regional and national scales, consistent with previous studies [[28], [29], [30], [31]]. Furthermore, our investigation revealed that the goodness of fit for Singapore and Brunei's carbon emission inversion models is significantly lower than that of the Southeast Asia model and models of other member countries. We attribute this discrepancy to limitations in the NTL data. Singapore and Brunei are economically developed countries with small land areas and high per-unit-area human-induced carbon emissions. Although these countries exhibit high NTL intensity indices, the calibration process constrains their values due to lower saturation thresholds. As a result, the models struggle to fully capture human-induced carbon emissions, leading to a lower goodness of fit in the constructed models.

Our model is designed to estimate carbon emissions in Southeast Asia based on NTL data and socio-economic indicators. However, the methodology is not region-specific and can be adapted to other regions with appropriate modifications. For instance, the model could be refined in industrialized regions like China and Europe by incorporating local industrial emissions inventories, detailed energy consumption datasets, and sectoral carbon intensity factors. Differences in economic structure and energy mix [52] would influence the model's output, necessitating region-specific calibration. Policy frameworks such as carbon pricing in the EU and regional emission trading schemes in China could be integrated into the model to reflect regulatory impacts better. Future research could explore these adaptations to validate our approach's generalizability further.

While this study and ODIAC employ NTL data for carbon emission estimation, key methodological differences distinguish our approach. Unlike ODIAC, which relies on global power plant data and fossil fuel consumption statistics, our model integrates economic metrics (e.g., GDP), human activity data, and land-use information to enhance regional estimation accuracy. We employ Polynomial Regression and machine learning (Random Forest, Support Vector Regression) to calibrate NPP-VIIRS and DMSP-OLS data, reducing saturation biases and improving long-term consistency. Additionally, trend analysis methods (Theil-Sen estimator, Mann–Kendall test) capture long-term carbon emission dynamics, whereas ODIAC primarily provides static distributions. Our approach refines emission estimates by incorporating socio-economic activity, enabling more granular differentiation between core cities and secondary urban areas—an aspect not explicitly addressed in ODIAC. These refinements make our model particularly valuable for regional-scale policy planning and low-carbon transition strategies in Southeast Asia.

### 4.3 Spatio-temporal dynamics and policy strategies for carbon emissions

From 1992 to 2022, Southeast Asia experienced rapid development, characterized by a spatial differentiation of human-induced carbon emissions. This pattern is marked by rapid growth in emissions in large cities and slow growth in small towns [53,54]. The uneven socio-economic development in Southeast Asia is evident, with resources heavily concentrated in key cities such as the capitals and major cities of Vietnam, Thailand, Malaysia, and Indonesia. This concentration places significant pressure on these cities' ecological environments and hinders their sustainable development [[55], [56], [57]]. Therefore, regional coordination and sustainable development are imperative for future top-level planning. Our study demonstrates consistent temporal and spatial trends through comparative analysis with ODIAC and EDGAR data products. In high-emission cities and regions within Southeast Asia, the correlation between our results and those from ODIAC and EDGAR regarding temporal and spatial trends is nearly identical. This correlation confirms the feasibility of our approach, which involves decomposing NTL unit grids to simulate human-induced carbon emissions.

Trend analysis and significance testing of human-induced carbon emissions in Southeast Asia from 1992 to 2022 reveal a “dominant cycle” trend in major cities such as Bangkok, Ho Chi Minh City, Hanoi, Kuala Lumpur, and Jakarta. This trend indicates that these cities, initially high-emission or critical areas, continue to display high emissions during socio-economic development [[58], [59], [60]]. Comparing carbon emissions and GDP during this period shows that high economic growth in the region is accompanied by high energy consumption and emissions (Fig. S5), highlighting Southeast Asia's relatively low quality of economic development. Initiatives such as “zero carbon emissions” and regional low-carbon economic strategies will constrain this “high growth and high emissions” pattern, necessitating a more precise strategy for future energy development, energy consumption, and production. This situation reflects an unreasonable regional socio-economic development plan, where the “siphon effect” of large cities impedes the development of small towns and leads to extensive development patterns. The “urban sprawl” expansion has established a deeply-rooted development model for these cities, posing challenges to implementing subsequent policies related to “zero carbon emissions” and related measures [61,62]. Based on the Hurst index combined with trend analysis, we predict that if the current development trend continues, human-induced carbon emissions in large cities will strengthen, and carbon emissions in surrounding areas will gradually increase. Meanwhile, small towns far from large cities will experience minimal changes in carbon emissions. Achieving regional development balance is crucial for sustainable development. Implementing top-level planning, balancing infrastructure construction across regions, optimizing resource allocation and energy patterns, and promoting circular and sustainable socio-economic development are vital approaches to addressing Southeast Asia's “high-emission, high-growth” phenomenon and the “dominant cycle” of emissions in major cities.

Although our study effectively captures the spatio-temporal evolution of carbon emissions across Southeast Asia, certain limitations must be acknowledged. This study primarily relies on NTL data to proxy for human-induced emissions. However, it may not fully capture industrial sources, particularly in energy-intensive sectors such as cement and steel production, which often operate outside urban cores [63]. Additionally, rural and peri-urban regions with low artificial illumination may have underestimated emissions, affecting the accuracy of regional comparisons [48]. These factors highlight the need for supplementary data sources to refine carbon emission estimates.

## 5 Conclusion

This study presents a spatial visualization model for estimating carbon emissions in Southeast Asia, integrating calibrated NTL data, economic metrics, and annual emission inventories. The findings reveal a 2.51-fold increase in emissions over 31 years, with rapid urbanization and industrial expansion as primary drivers. To effectively transition toward a low-carbon economy, we propose three region-wide strategies: (1) Decarbonizing urban-industrial hubs: Implement low-emission zones and congestion pricing in megacities like Bangkok, Manila, and Jakarta to curb transport-related emissions. Accelerate renewable energy adoption in industrial parks across Vietnam, Malaysia, and Thailand by mandating solar and wind power integration, aligning with ASEAN's 35% renewable energy target by 2035. (2) Establishing a regional carbon market and industrial emission caps: Introduce an ASEAN-wide emissions trading system (ETS), leveraging Singapore's pilot scheme as a model. Enforce stricter emission caps in high-emission industries, such as cement production in Indonesia and steel manufacturing in Vietnam, while incentivizing carbon capture, utilization, and storage (CCUS) in petrochemical sectors. (3) Advancing circular economy models in manufacturing and agriculture: Develop zero-waste industrial clusters in Malaysia's electronics sector and Thailand's automotive supply chains, promoting resource efficiency and waste-to-energy innovations. Expand sustainable agriculture by implementing methane capture from rice paddies in the Mekong and Irrawaddy deltas, reducing agricultural emissions while enhancing food security. Additionally, it will strengthen carbon monitoring by integrating AI-driven tracking and satellite-based enforcement. These strategies, informed by our study's insights, offer a comprehensive roadmap for Southeast Asia's sustainable economic transformation.

## CRediT authorship contribution statement

**Chaoqing Huang:** Writing – review & editing, Writing – original draft, Software, Methodology, Data curation, Conceptualization. **Qian Wu:** Visualization, Validation, Data curation. **Yujie Chen:** Visualization, Validation, Data curation. **MinhThu Nguyen:** Software, Methodology. **Bin Chen:** Software, Methodology. **Song Hong:** Writing – review & editing, Supervision, Conceptualization. **Chao He:** Writing – review & editing, Supervision, Methodology, Funding acquisition, Conceptualization.

## Data availability statement

The data used to support the results of this research are shown in the manuscript and available from the corresponding author upon request.

## Declaration of competing interests

The authors declare that they have no known competing financial interests or personal relationships that could have appeared to influence the work reported in this paper.
