# Supplementary material for: Spatio-temporal dynamics of human-induced carbon emissions in Southeast Asia (1992–2022) based on nighttime light
Source: Eco Environ Health. 2025 Apr 26;4(2):100150. doi: 10.1016/j.eehl.2025.100150 (PMC12136777; doi:10.1016/j.eehl.2025.100150)
Supplement: Multimedia component 1 [file mmc1.docx]

## Supplementary material

**Spatio-temporal dynamics of human-induced carbon emissions in Southeast Asia (1992–2022) based on nighttime light**

Chaoqing Huang ^a,b^, Qian Wu ^a,b^, Yujie Chen ^c^, MinhThu Nguyen ^d^, Bin Chen ^e^, Song Hong ^a,b,*^, Chao He ^f,g,*^

^a^ School of Resource and Environmental Sciences, Wuhan University, Wuhan 430079, China

^b^ Key Laboratory of Geographic Information System, Ministry of Education, Wuhan University, Wuhan 430079, China

^c^ School of Electronic Information and Communications, Huazhong University of Science and Technology, Wuhan 430074, China

^d^ Vietnam Institute of Meteorology Hydrology and Climate Change, Ministry of Natural Resources and Environment, Hanoi 100803, Vietnam

^e^ Future Urbanity & Sustainable Environment (FUSE) Lab, Division of Landscape Architecture, Department of Architecture, Faculty of Architecture, The University of Hong Kong, Hong Kong 999077, China

^f^ National Science Library (Wuhan), Chinese Academy of Sciences, Wuhan 430071, China

^g^ Collaborative Innovation Center for Emissions Trading System Co-constructed by the Province and Ministry, Wuhan 430205, China

^*^ Corresponding authors.

E-mail addresses: chao_ho@163.com (C. He), songhongpku@126.com (S. Hong).

**Table S1**

Dataset.

| **Parameter** | **Dataset** |
| --- | --- |
| Nighttime light | NOAA/DMSP-OLS/NIGHTTIME_LIGHTS ^a^ (1992-2013) |
| Nighttime light | NOAA/VIIRS/001/VNP46A2 ^b^ (2013-2022) |
| CO_2_ emissions | Total CO_2_ emissions ^c^ (1992-2020) |
| CO_2_ emissions | ODIAC2020b ^d^ (2000-2019) |
| CO_2_ emissions | EDGAR v7.0 ^e^ (1992-2021) |
| CO_2_ emissions  Economic data | [CO](https://data.worldbank.org/indicator/EN.ATM.CO2E.PC?locations=ID)_[2](https://data.worldbank.org/indicator/EN.ATM.CO2E.PC?locations=ID)_ [emissions](https://data.worldbank.org/indicator/EN.ATM.CO2E.PC?locations=ID) ^f^ (1992-2021)  GDP ^f^ (1992-2021) |
| Economic data | GDP ^g^ (1992-2022) |
| Land cover | MCD12Q1 ^h^ (2013) |

Data Source：

a. https://developers.google.com/earth-engine/datasets/catalog/NOAA_DMSP-OLS_NIGHTTIME

_LIGHTS

b. https://developers.google.com/earth-engine/datasets/catalog/NOAA_VIIRS_001_VNP46A2

c. https://www.iea.org/data-and-statistics/data-tools

d. https://db.cger.nies.go.jp/dataset/ODIAC/

e. https://edgar.jrc.ec.europa.eu/

f. https://data.worldbank.org/

g. https://www.imf.org/en/Data

h. https://developers.google.com/earth-engine/datasets/catalog/MODIS_061_MCD12Q1

All carbon dioxide emissions are converted into carbon emissions using a conversion factor of 12/44.

**Table S2**

Models for calibration of NPP/VIIRS to DMSP/OLS data thresholds.

| Model | R² | RMSE |
| --- | --- | --- |
| Linear Regression | 0.33 | 19.02 |
| Polynomial Regression | 0.46 | 19.28 |
| Exponential Regression | 0.86 | 2.67 |
| Random Forest Regression | 0.80 | 10.05 |
| Support Vector Regression | 0.85 | 8.67 |
| K-Nearest Neighbors Regression | 0.83 | 9.24 |

**Table S3**

Performance comparison of polynomial regression, random forest, and support vector regression models in estimating carbon emissions for SEA countries/regions.

| Countries/ Regions | Polynomial Regression | | Random Forest | | SVR | |
| --- | --- | --- | --- | --- | --- | --- |
|  | R^2^ | RMSE | R^2^ | RMSE | R^2^ | RMSE |
| INDO | 0.95 | 9.09 | 0.47 | 21.86 | 0.29 | 25.32 |
| MALA | 0.96 | 5.35 | 0.57 | 8.24 | 0.66 | 7.28 |
| VIET | 0.97 | 2.04 | 0.71 | 12.19 | 0.31 | 18.60 |
| THAI | 0.95 | 4.48 | 0.72 | 5.30 | 0.77 | 4.84 |
| PHI | 0.95 | 1.39 | 0.71 | 3.66 | 0.49 | 4.86 |
| LAO | 0.86 | 0.75 | 0.83 | 0.72 | 0.78 | 0.83 |
| BRU | 0.74 | 0.16 | 0.47 | 0.23 | 0.84 | 0.13 |
| CAM | 0.95 | 0.16 | 0.78 | 0.49 | 0.95 | 0.24 |
| MYAN | 0.90 | 0.46 | 0.66 | 1.31 | 0.70 | 1.23 |
| SIN | 0.75 | 0.70 | 0.05 | 0.64 | 0.02 | 0.64 |
| SEA | 0.99 | 21.10 | 0.51 | 59.81 | 0.06 | 82.62 |

**
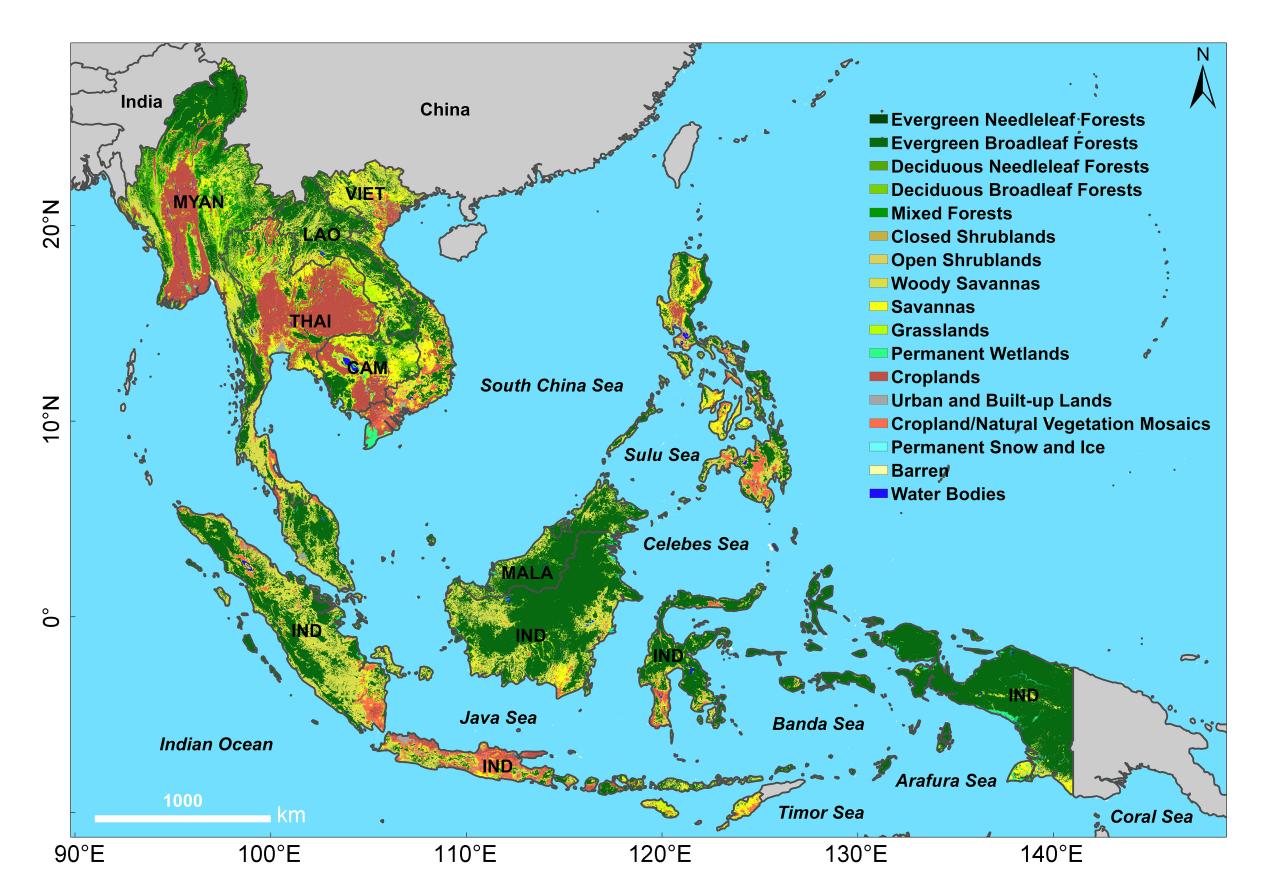
**

**Fig. S1.** Land Cover Map of Southeast Asia in 2013.


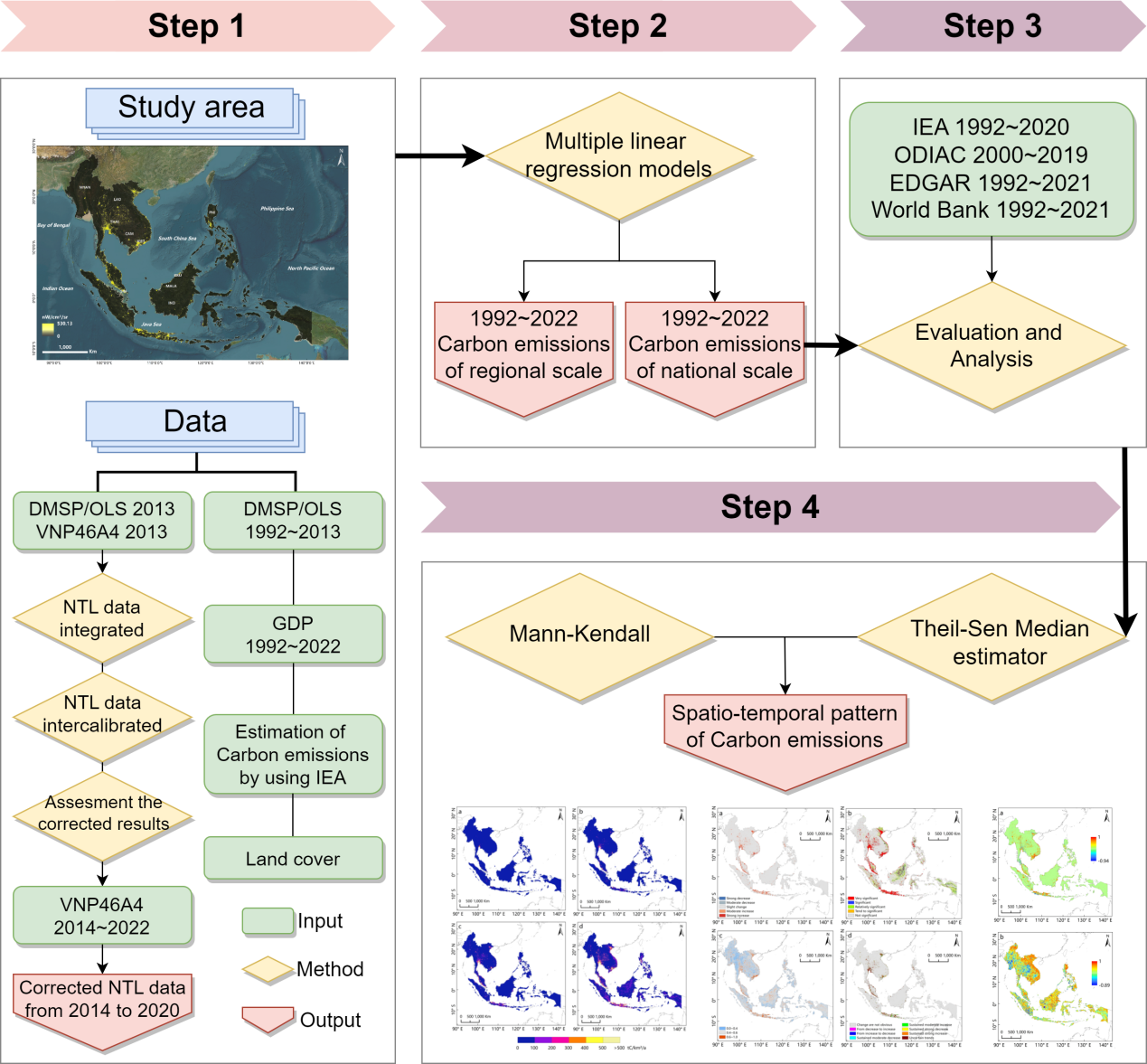


**Fig. S2.** The flow chart of spatio-temporal dynamics and modeling of carbon emissions in Southeast Asia (1992-2022).


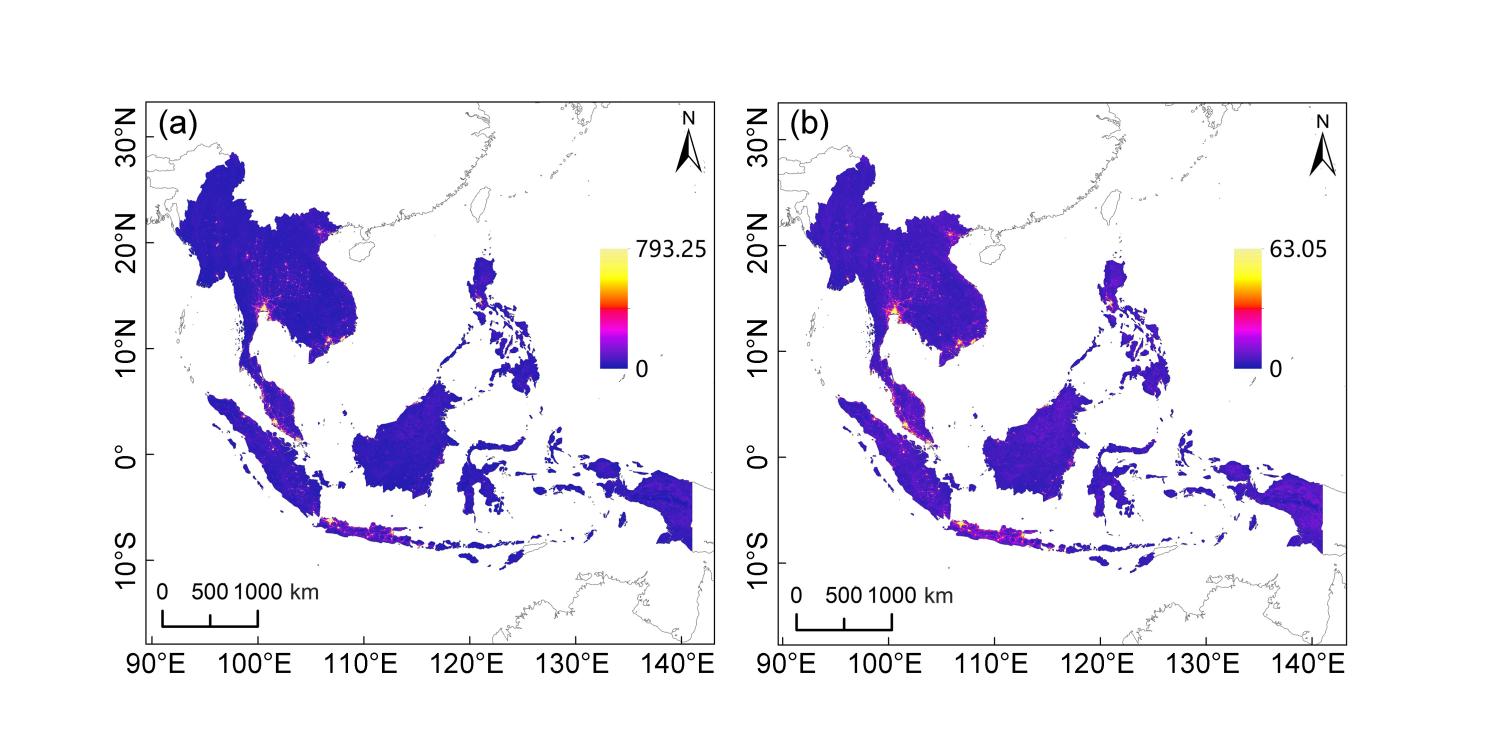


**Fig. S3.** NPP/ VIIRS 2013 Nighttime light, before calibration (a), after calibration (b).


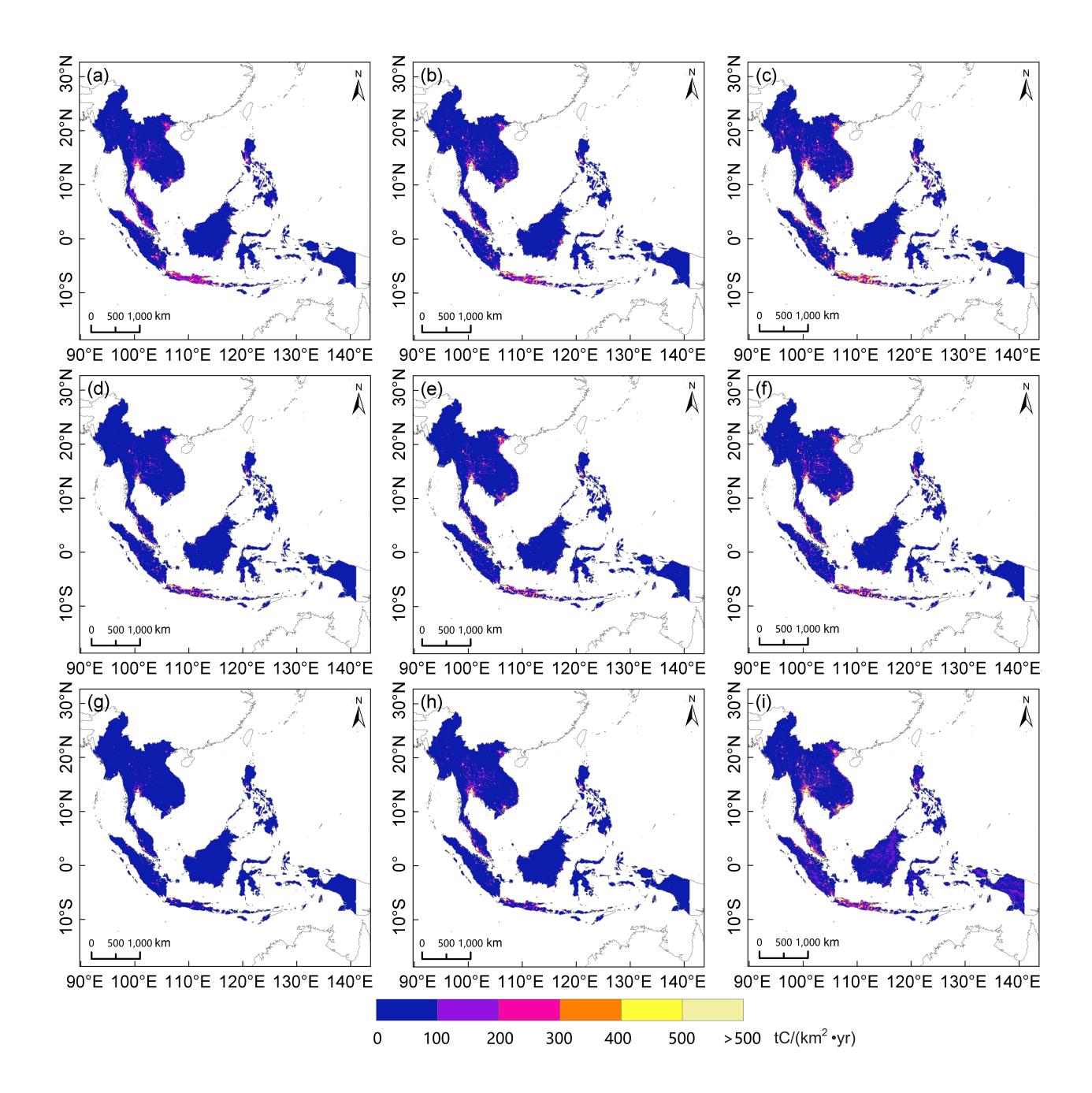


**Fig. S4.** Human-induced carbon emissions in Southeast Asia, ODIAC2001(a), ODIAC 2010(b), ODIAC 2019(c), EDGAR2001(d), EDGAR 2010(e), EDGAR 2019(f), this study2001(g), this study2010(h), this study2019(i).


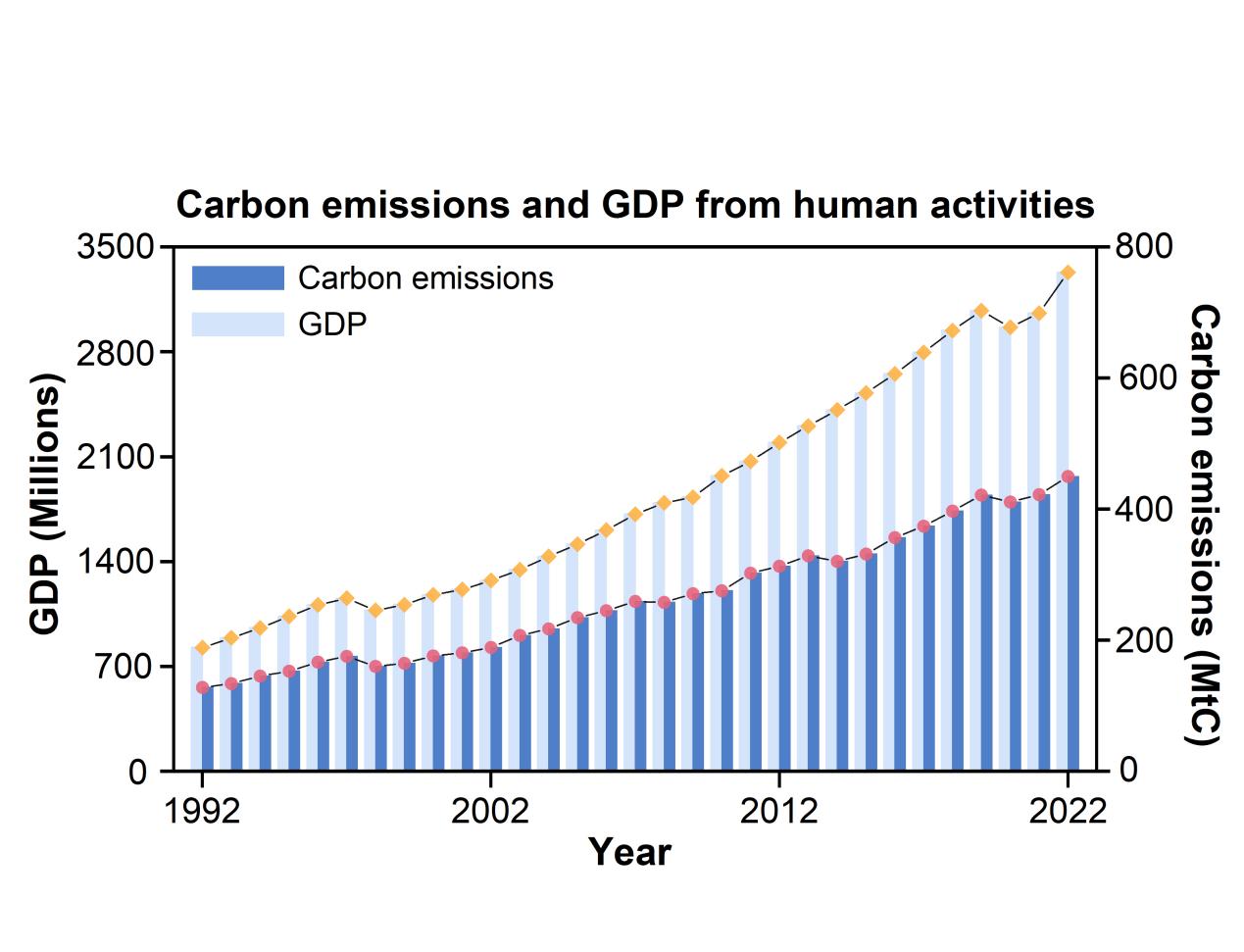


**Fig. S5.** Trends of carbon emissions and GDP from human activities in Southeast Asia from 1992 to 2022.
